# The intersection and developmental trajectory of morning cortisol and testosterone in autistic and neurotypical youth

**DOI:** 10.1186/s13229-025-00658-0

**Published:** 2025-04-24

**Authors:** Blythe A. Corbett, Trey McGonigle, Rachael A. Muscatello, Simon Vandekar, Rachel Calvosa

**Affiliations:** 1 Department of Psychiatry and Behavioral Sciences, Vanderbilt University Medical Center, 1500 21st Avenue South, Nashville, TN 37212 USA; 2 Vanderbilt University Medical Center, Vanderbilt Kennedy Center, Nashville, TN USA; 3 Department of Psychology, Vanderbilt University, Nashville, TN USA; 4 Department of Biostatistics, Vanderbilt University Medical Center, Nashville, TN USA

**Keywords:** Autism, Puberty, Testosterone, Cortisol, Hormones, HPA axis, HPG

## Abstract

**Background:**

Behavioral endocrinology examines associations between hormone expression, such as testosterone and cortisol, and behavior; both of which have been implicated in autism spectrum disorder (ASD). The overarching aim of the study was to examine the intersection of sex-based (Male, Female), hormonal (testosterone, cortisol), diagnostic (ASD, typically developing, (TD)) and developmental (age, puberty) patterns over four years of a longitudinal study in a well-characterized sample of youth (spanning 10 to 17 years).

**Methods:**

In year 1 (Y1), participants included 140 autistic youth (36 females, 104 males) and 105 TD youth (46 females, 59 males.). For Y4, participants included 83 ASD and 77 TD youth. Immediate waking morning salivary samples were collected for hormone assay. Mixed effects and ordinary linear regression models were used, as well as mediation effects of hormones on behavior.

**Results:**

For cortisol, there was a significant diagnosis by sex by age interaction (X^2^ = 15.62, df = 3, *p* = 0.0014, *S* = 0.2446) showing that autistic females evidence higher morning cortisol that increased over developmental progression compared to TD females. Moreover, ASD males had stunted testosterone growth compared to TD males (Est = 0.1530, *p* = 0.0130). Regarding biobehavioral associations in year 1, diagnosis (X^2^ = 80.72, df = 1, *p* < 0.0001, *S* = 0.5704) and cortisol (X^2^ = 14.42, df = 3, *p* = 0.0024, *S* = 0.2159) were associated with social problems; however, there were no effects for testosterone on diagnosis or a mediation effect on social problems. There was a significant effect of diagnosis on CBCL Aggression score (X^2^ = 34.39, df = 1, *p* < 0.0001, *S* = 0.3692) independent of hormonal measurements.

**Limitations:**

Despite the large sample, it was not fully representative based on race, ethnicity or intellectual profile. Attrition of the sample is also acknowledged especially between portions of Y2 and Y3 due to the COVID-19 pandemic. Finally, only the immediate morning salivary samples were used due to lower and undetectable concentration levels of testosterone in younger and female children.

**Conclusions:**

Collectively, these findings underscore the need to elucidate the biobehavioral patterns that emerge during the complex adolescent transition for autistic youth to determine how they impact clinical and long-term outcomes. The unique hormonal trajectories may be related to differences in advanced pubertal progression and affective states found in autistic females.

**Supplementary Information:**

The online version contains supplementary material available at 10.1186/s13229-025-00658-0.

## Background

Autism spectrum disorder (ASD) is clinically defined by challenges in reciprocal social communication as well as restricted, repetitive and stereotyped behavior [1]. Many autistic individuals experience significant difficulty with novelty and adapting to change [2] which include developmental transitions such as adolescence [1, 3]. Indeed, adolescence has been proposed as a period of heightened vulnerability in autism [4].

Epidemiological studies of autism have generally reported a diagnostic bias of a 4:1 male-to-female ratio [5]; yet, some research suggests the ratio may be closer to 3:1 ratio [6] due to a more subtle female phenotype which presents with reduced social challenges and repetitive behaviors (e.g [7–9]). Importantly, a variety of sex-based differences have been found in terms of mental health, social communication, masking behavior and physiological profiles (e.g [7, 10–12]). Differential patterns are particularly relevant during the adolescent years.

### Adolescence

Adolescence is the bridge between childhood and adulthood thereby it is a period of remarkable changes in social and cognitive functioning [13]. It covers a broad age range from the early stage (10–14 years) to middle (15–17 years) to late adolescence and early adulthood (17–24 years) [14]. Puberty, which often parallels adolescence, explicitly refers to the hormonal, physiological and physical development patterns resulting in primary and secondary sexual characteristics (e.g [13]). Pubertal onset varies broadly depending on demographic, biobehavioral and environmental factors [15]. Deviations in pubertal onset have been shown to negatively impact mental well-being especially early onset for females and late onset for boys [16]. In fact, advanced pubertal onset in females has been associated with higher rates of psychological distress [17] and depression (e.g [18–21]). Autistic females have been shown to experience early onset puberty potentially predisposing them to biopsychosocial risk [22, 23].

### HPA axis and HPG axis

The Hypothalamic-Pituitary-Adrenal (HPA) axis is a neuroendocrine system involved in many key regulatory processes including development, homeostasis and adjusting the intricate balance of hormones in response to stress. In humans, cortisol is characterized by a circadian rhythm with high concentrations in the morning, decline throughout the day, and low concentrations in the evening generally corresponding with routine patterns of light and activity. During adolescence, the HPA axis undergoes physiological changes including higher basal levels of the glucocorticoid cortisol [24], a flatter diurnal slope [25], and increased cortisol responsivity to perceived stress [26]. There are also sex-based differences during adolescence with females exhibiting higher basal cortisol and stronger circadian rhythm compared to same-age males [25, 27]. Such differences in cortisol may be associated with higher rates of adolescent-onset mental health conditions in females, such as depression [28]. Moreover, it is well-established that HPA axis regulation is affected by metabolic factors (e.g., body mass index (BMI) (e.g [25, 27, 29]), and use of some pharmaceutical agents [30].

The Hypothalamic-Pituitary-Gonadal (HPG) axis is essential to the onset of puberty, sexual maturation, and secretion of gonadal hormones including testosterone and estradiol (e.g [31, 32]). The HPG becomes active during three key periods: early gestation [33], postnatal development [34–36] and pubertal onset [37]. Hormones produced by the HPG axis, including testosterone play a pivotal role in both the brain and periphery impacting neuronal growth and migration [32], as well as the expression of sex-specific social behaviors [38]. As such, HPG activation influences physical, physiological and psychological functioning.

### Behavioral endocrinology

Behavioral endocrinology examines the association between hormone expression and behavior. Robust examples can be found in human and animal research focused on the influence of testosterone and cortisol on behavior. For example, links have been made showing associations between levels of testosterone and competition [39], dominance [40], aggression [41] and risk-taking behavior [42]. Additionally, a plethora of research has shown relationships between cortisol levels and perceived stress (e.g [43, 44], see meta-analysis review [45]).

Furthermore, the HPG and HPA axis and their end products; namely, testosterone and cortisol, interact on several neurobiological levels to jointly regulate behavior [46]. One postulated interaction, known as the “Dual Hormone” hypothesis, proposes that higher testosterone is positively associated with status-seeking tendencies especially when cortisol is low [39]. In other words, cortisol may play a moderating role and block the influence of testosterone, which has garnered support on aspects of dominance, leadership and status-striving behaviors in studies conducted in adults [47–49]. However, findings are mixed with some supporting the dual-hormone hypothesis [48] whereas others show no robust interaction [50]. Since most studies examining this hypothesis have been conducted in adults, it is unclear the extent to which the proposed hormone relationship may be applicable in adolescents. With that said, it is relevant to highlight that the HPA and HPG and associated hormones have been implicated in ASD.

Furthermore, the prevalence of aggression in children and adolescents with ASD is high with reports of aggression ranging from 49 to 68% toward non-caregivers to caregivers, respectively [51]. In a large cohort of children and adolescents ages 7 to 17 years with ASD (*N* = 450) and TD (*N* = 432) showed autistic youth exhibited significantly more verbal aggression compared to same age peers [52] and inflexibility observed in ASD has also been predictive of aggression. As such, examining hormone profiles in relation to aggressive behavior is of interest.

### HPA axis, cortisol and ASD

Research in salivary cortisol in children and adolescents with autism has frequently reported dysregulation of the HPA axis showing variable diurnal rhythms [53–57], which includes elevated evening cortisol [55, 58] and blunted diurnal slope [59, 60]. The diminished cortisol slopes were replicated in a recent large, longitudinal study revealing that, in addition to a diagnosis of ASD, age, puberty, and sex can play a role [58]. In some studies patterns of diurnal cortisol have been less reliable (for review see [61]). For example, findings for the cortisol awakening response have been mixed [62–64]. Similarly, some children with ASD and intellectual disability have shown higher mean cortisol elevations [59, 60], whereas others have shown no significant diurnal differences [65]. Cortisol is involved in stress responsivity and several studies have shown significant elevations in cortisol response to benign social interaction with peers [55, 66, 67] yet blunted cortisol response to social evaluative threat in youth (e.g [68–73]). The heightened cortisol during social interaction have also been shown to increase with age in a sample of children 8 to 12 years [74] and over pubertal development [75]. Taken together, previous research has shown atypical cortisol regulation and responsivity in autism.

### HPG axis, testosterone and ASD

The important role of testosterone in prenatal development and neural organizing effects has led to speculation of associations between androgens and the development of autism. The prenatal steroid theory (previously coined *fetal androgen theory*) postulates that autism may be the result of exposure to elevated levels of androgens during fetal development (e.g [76–79]). However, others have found no relationship between prenatal androgen exposure (e.g., testosterone) and ASD or autistic traits [80–83]. Yet most of the research in autism and testosterone has been conducted early in development. Literature in pre-pubertal samples have demonstrated elevated androgens (e.g., testosterone, dehydroepiandosterone) in youth with ASD relative to neurotypical controls [84–87]. But other studies in prepubertal samples have not found associations with androgens [88] or even lower testosterone [89] in ASD. In consideration of hormonal changes associated with adolescence and pubertal development, a recent study utilizing the current sample of 244 adolescents (ASD = 144, TD = 104) reported that higher morning testosterone levels were shown in autistic youth (aged 10 to 13 years) compared to neurotypical youth suggesting that it may play an influential role in ASD during developmental periods such as pubertal progression [90]. In addition to diagnostic and developmental factors, sex-based differences in testosterone were also noted.

Although research has shown differences in hormone expression in autistic compared to neurotypical individuals, little research has examined the interplay between cortisol and testosterone within the same participants. One study examined the hormone levels related to arousal/stress (cortisol), arousal/aggression (testosterone), and social/affiliation (oxytocin) and the extent to which they were related to aggression and callous-unemotional characteristics in adolescents with ASD, TD or oppositional defiant/conduct disorder [91]. While hormone expression differed across the groups, relationships were generally not significant and the method of hormone sampling (e.g., not measured at the same time of day, only one timepoint) limited the robustness of the findings.

The current 4-wave longitudinal study described below, can more thoroughly and rigorously examine this complex developmental period to reveal patterns based on diagnostic group, biological sex, and hormonal profiles. While we look for potential changes based on these factors, it is also meaningful to discover that some consistency exists across these important determinants.

### Current study

The overarching aim of the study was to examine the intersection of sex-based (Male, Female) hormonal (testosterone, cortisol), diagnostic (ASD, TD) and developmental (age, puberty) patterns over four years of a longitudinal study in a well-characterized sample of youth (spanning 10 to 17 years). The aims and hypotheses (Hyp) follow. Aim 1: to examine the developmental trajectory of cortisol and testosterone based on sex (Male, Female) and diagnosis (ASD, TD) over development (Age, Puberty). Hyp 1: Developmental effects were predicted such that cortisol and testosterone would increase over adolescence (age) and puberty (Tanner stage).

Aims 2 and 3 investigate the mediating effects of cortisol and testosterone on behavioral issues in early and late pubertal development. Aim 2: to investigate the joint mediation effect of cortisol and testosterone on diagnosis’ effect on the manifestation of social problems. Hyp 2: It was hypothesized that diagnostic effects on CBCL Social Problems (CBCL-SP; [92]) are driven by differences in the cortisol and testosterone profile. Aim 3: to evaluate the Dual-Hormone Hypothesis in explaining aggressive behavior by differences in hormone profiles. Hyp 3: It was hypothesized that diagnostic effects on CBCL Aggression (CBCL-A; [92]) are mediated by differences in the cortisol and testosterone profile.

## Methods

### Participants

The data for the current study was collected as part of a large, longitudinal study on pubertal development and stress [93]. The study includes data from all four years of the assessment years: Year-1 (Y1) enrollment occurred when the children were between 10-years-0-months to 13-years-11-months of age. Subsequent visits occurred annually. Participants were recruited from the southern United States covering a 200-mile radius that targeted medical and health-related services, clinics, research registries, regional disability organizations, schools, and social media platforms. Inclusion required an intelligence quotient (IQ) score *≥* 70 due to task demands in the source longitudinal study. Children were excluded if taking medications that alter the Hypothalamic-Pituitary-Adrenal (HPA) axis (e.g., corticosteroids; see [30]) or HPG axis (e.g., growth hormone, oral contraceptives, nicotine), known to influence the HPA or HPG [94, 95]. Additionally, medical conditions that may impact pubertal development, such as Cushing’s Disease, were exclusionary. Regarding medication status, in the ASD group 65.2% of were taking at least one medication compared to 17.5% in the TD group. Across the sample, medication use included stimulants, selective-serotonin reuptake inhibitors, melatonin, antihistamines, and central alpha-agonists.

In Y1, there were 245 youth (239 participants that completed the physical exam described below). The ASD group consisted of 140 participants (median age 11.2 years) including 36 females and 104 males. The TD group consisted of 105 participants (median age 11.7 years) including 46 females and 59 males. One autistic male was missing a measurement for the physical exam stage.

In Y2 there were 183 participants, the ASD group had a median age of 12.5 years, and the TD group had a median age of 12.7 years. The overall attrition rate was 25.31%, which was comparable to other longitudinal studies after the initial enrollment [96]. At Y3, there were 169 participants, with a median age of 13.4 years for the ASD group and 13.8 years for the TD group. At Y4, there were 160 participants (median age 14.4 years for ASD and 14.6 years for TD group). At Y2 and Y3, some participants were unable to complete the full physical examination due to restrictions on in-person lab visits resulting from the COVID-19 pandemic (Y2 *N* = 43; Y3 *N* = 59).

At Year 1, the racial and ethnic characterization of the sample included 7.8% Black, 83.3% White, and 8.6% multiracial. Demographic information for each group is presented in Table 1. Importantly, it should be noted that a longitudinal analysis of diurnal cortisol [58] and a report of testosterone concentrations at Year 1 [90] have been previously published for the sample and have been appropriately cited and acknowledged throughout. All cross-sectional and longitudinal analyses of morning cortisol and testosterone in relation to the Dual Hormone hypothesis, as well as examination of relationships with social problems or aggressive behaviors, are novel and have not been published previously for the current sample.

**Table 1:** Descriptive and demographic statistics stratified by diagnosis

	TD	ASD	Overall	*p*-value
	(*N* = 105)	(*N* = 140)	(*N* = 245)	
Age (Year 1)	10.58 **11.67** 12.67	10.50 **11.25** 12.25	10.58 **11.33** 12.33	0.081^1^
Age (Year 2)	11.76 **12.74** 13.82	11.70 **12.46** 13.52	11.73 **12.65** 13.62	0.245^1^
Age (Year 3)	12.75 **13.68** 14.79	12.68 **13.37** 14.43	12.74 **13.53** 14.51	0.183^1^
Age (Year 4)	13.75 **14.59** 15.56	13.75 **14.42** 15.40	13.74 **14.48** 15.48	0.549^1^
Change in Age (Y1 – Y4)	3.13 **3.18** 3.27	3.14 **3.18** 3.30	3.13 **3.18** 3.28	0.491^1^
Sex				0.003^2^
Male	0.56 59/105	0.74 104/140	0.67 163/245	
Female	0.44 46/105	0.26 36/140	0.33 82/245	
Ethnicity				0.332^2^
Not Hispanic	0.95 100/105	0.92 129/140	0.93 229/245	
Hispanic	0.05 5/105	0.08 11/140	0.07 16/245	
Race				0.002^3^
Caucasian	0.86 90/105	0.81 114/140	0.83 204/245	
African American	0.02 2/105	0.12 17/140	0.08 19/245	
American Indian	0.00 0/105	0.00 0/140	0.00 0/245	
Asian/Pacific Islander	0.00 0/105	0.01 1/140	0.00 1/245	
Biracial	0.12 13/105	0.06 8/140	0.09 21/245	
BMI (Percentile, Year 1)	30.00 **53.00** 87.00	39.25 **69.00** 95.75	34.00 **63.00** 93.00	0.014^1^
GB Development (Year 1)	1.00 **2.00** 3.00	1.00 **2.00** 3.00	1.00 **2.00** 3.00	0.835^1^
GB Development (Year 2)	2.00 **2.00** 4.00	2.00 **3.00** 4.00	2.00 **3.00** 4.00	0.250^1^
GB Development (Year 3)	2.00 **4.00** 5.00	3.00 **4.00** 5.00	3.00 **4.00** 5.00	0.824^1^
GB Development (Year 4)	4.00 **4.00** 5.00	4.00 **5.00** 5.00	4.00 **5.00** 5.00	0.533^1^
GB Development (Y1-Y4)	2.00 **2.00** 3.00	1.00 **2.00** 3.00	1.00 **2.00** 3.00	0.105^1^
Cortisol (Year 1)	6.90 **9.09** 12.16	6.11 **8.02** 10.92	6.39 **8.55** 11.41	0.056^1^
Cortisol (Year 4)	6.55 **9.03** 12.32	6.14 **8.66** 10.94	6.40 **8.81** 11.37	0.270^1^
Testosterone (Year 1)	0.00 **0.00** 0.01	0.00 **0.01** 0.03	0.00 **0.01** 0.02	0.183^1^
Testosterone (Year 4)	0.02 **0.07** 0.13	0.03 **0.10** 0.17	0.02 **0.09** 0.15	0.202^1^

Note ^1^ Wilcoxon Rank Sum Test, ^2^ Chi-Square Test of Homogeneity, ^3^ Fisher’s Exact Test; Q1 **Median** Q3; Cortisol, nmol/L; Testosterone, ng/mL

GB, Genital/Breast

The research was carried out in accordance with the Code of Ethics of the World Medical Association (Declaration of Helsinki). The Vanderbilt Institutional Review Board approved the study. Informed written consent and assent was obtained from all parents and study participants, respectively, prior to inclusion in the study.

The diagnosis of ASD was confirmed based on the Diagnostic and Statistical Manual-5 [1] and established by: (1) a diagnosis by a psychologist, psychiatrist, or behavioral pediatrician with autism expertise; (2) current clinical judgment, and (3) corroborated by the Autism Diagnostic Observation Schedule (ADOS-2; [97]), which was administered by research-reliable clinicians.

#### Diagnostic measures

The diagnostic measures were administered during Y1 of the study.

***Autism Diagnostic Observation Schedule-Second Edition*** (ADOS-2; [97]) is a semi-structured interactive play and interview-based instrument used to support the diagnosis of ASD. The ADOS Module III was administered by research-reliable personnel. A score of 7 or above is consistent with a diagnosis of ASD.

***Social Communication Questionnaire*** (SCQ; [98]) is a screening questionnaire to assess for symptoms of ASD. A score of 15 is suggestive of a diagnosis of ASD. Due to lower sensitivity and specificity [99], TD children with a score *≥* 10 were excluded from the study.

***Wechsler Abbreviated Scale of Intelligence***,*** Second Edition*** (WASI-II [100]), a measure of cognitive ability, was used to obtain an estimate of the participant’s intellectual functioning. Inclusion for the study required an IQ *≥* 70.

#### Dependent measures

The dependent measures were administered annually each year of the study (Y1 – Y4).

***Physical Examination.*** The gold standard physical exam was completed at each annual visit to reliably identify pubertal development and assign Tanner stage [101, 102], which assigns two measures with 5 stages from 1 (not begun) to 5 (fully developed) for Male External Genitalia (G1-G5 for males) and Female Breast (B1-B5 for females) (G/B stage) and Pubic hair (P1-P5 for both sexes) (PH stage). Exams were conducted by trained, licensed study physicians and consisted of visual inspection. A full description of the physical examination procedures, including physicians’ reliability, can be found in [23].

***The Child Behavior Checklist*** (CBCL; [92]) is a broad-based parent report form used to provide children’s competencies and behavioral and emotional problems from 6 to 18 years of age. Items are presented on a Likert scale ranging from 0 (“Not True”) to 2 (“Very true”), such that higher scores reflect more symptom severity, and T-scores *≥* 70 indicate a clinically significant symptom profile. The CBCL Social Problems (CBCL-SP) and Aggressive Behavior (CBCL-A) scales were used. The CBCL Social Problems (*r* = 0.90) and Aggressive Behavior (*r* = 0.90) scales both have high test-retest reliability. The construct validity of CBCL-A (*p* < 0.001, *r* = 0.72) and CBCL-SP (*p* < 0.001, *r* = 0.57) are significant, with a moderate correlation between CBCL-A score and its equivalent Behavior Assessment System for Children (BASC) score [103], and a modest correlation between CBCL-SP score and its equivalent BASC score [92]. The CBCL Social Problems scale (along with Attention and Thought scales) has been shown to differentiate children with ASD from TD children [104]. The CBCL was administered during the physical exam visit.

### Cortisol sampling

Morning saliva samples were collected annually at home as part of a diurnal sampling regime following natural waking [105–107] using established procedures (e.g., passive drool, postponed if sick) [53, 54]. Families and participants were thoroughly trained on collection procedures, including instructional materials and demonstration. Families methodically documented sampling day and time using daily diaries and recorded the collection time on sample labels. Diaries included prompts for recording time to bed, time woken, total sleep, and any important notes about the day. Per the protocol, participants passively drooled into a test tube using a straw collecting approximately 1 mL of saliva. Two autistic children were unable to utilize the passive drool method thus used a cotton roll and syringe procedure for their samples [60]. Sensitivity analyses excluding the two children revealed no meaningful difference; therefore, the participants’ data were included in the full dataset. Participants were instructed to not eat or drink for 1-hour prior to sample collection and to refrain from brushing teeth in the morning until after sample collection. Families were instructed to collect samples during the three weekdays prior to the lab visit. Samples were refrigerated in the home until returning to the lab at which time they were placed in a -80 °C freezer. To account for hormonal changes throughout the menstrual cycle, female participants that had begun menstruating were scheduled during the luteal phase, based on date of last menses, as previous research has shown women in the luteal phase to have comparable cortisol levels to men [108].

### Cortisol assay

Cortisol assays were performed using a Coat-A-Count^®^ radioimmunoassay kit (Siemens Medical Solutions Diagnostics, Los Angeles, CA) modified to accommodate lower levels of cortisol in human saliva. Samples stored at -80 °C, were thawed and centrifuged at 3460 rpm for 15 min to separate the aqueous component from mucins and other suspended particles. The coated tube from the kit was substituted with a glass tube into which 100 ul of saliva, 100 ul of cortisol antibody (courtesy of Wendell Nicholson, Vanderbilt University, Nashville, TN), and 100 ul of ^125^I-cortisol were mixed. After incubation at 4 °C for 24 h 100 ul of normal rat serum in 0.1% PO4/EDTA buffer (1:50) and precipitating reagent (PR81) were added. The mixture was centrifuged at 3460 rpm for 30 min, decanted, and counted (for details see [109]). Serial dilution of samples indicated a linearity of 0.99. The intra-assay coefficients of variation were as follows Y1 CV = 2.06%; Y2 CV = 2.80%; Y3 CV = 1.90%; Y4 CV = 2.07%. The total intra-assay (across all three years) = 2.19%. The inter-assay CV was 9.52%. Cortisol is reported in units of nmol/L.

### Testosterone sampling

Salivary testosterone can be measured reliably and non-invasively using small amounts of saliva, making it an ideal measure in studies of children and youth (e.g [110, 111]). Like cortisol, testosterone also shows a diurnal rhythm with levels highest in the morning and declining throughout the day [110]. Due to the young age of the participants, diurnal concentrations in the afternoon and evening were often below the assay’s level of detection (2.5 pg/ml), which is common in research especially in younger children [112, 113]. Therefore, only the immediate morning samples were used for the current study.

The testosterone samples were collected annually at the same time as the cortisol samples described above. If the participant became ill, home sampling was rescheduled until after the participant was healthy. For females, the menstrual cycle was documented, and basal testosterone was collected during the early Luteal phase to reduce variability across the menstrual cycle [114].

### Testosterone assay

The salivary testosterone radioimmunoassay (RIA) performed in Hormone Assay and Analytical Services Core Laboratory of the Vanderbilt Diabetes Research and Training Center was developed in the laboratories of the Division of the Diabetes, Endocrinology and Metabolism, Department of Medicine, Vanderbilt University Medical Center, Nashville, TN, 37232. The primary antibody to testosterone was purchased from MP Biomedicals, Cat#: 07-189113. Testosterone-19-Carboxymethlyether-BSA was used as the antigen to generate antiserum in rabbits. The antibody is highly specific to testosterone. Cross reactivity in the testosterone biosynthetic pathway is 5α-Dihydrotestosterone (3.4%), 5α-Androstane-3β,17β-diol (2.2%), 11-Oxotestosteron (2.0%), 6β-Hydroxytestosterone (0.95%), androstenedione (0.56%), progesterone (< 0.01%), and Estradiol-17β (< 0.01%).

The assay is performed with testosterone I-125 from MP Biomedicals, Cat: #07-189121. Prior to assay, saliva was stored at -20ºC, then thawed and centrifuged at 3460 rpm (2650 g) for 15 min to separate the aqueous component from mucins and other suspended particles. All samples were run in duplicate. The sensitivity of the assay is 0.0025 ng/ml. The inter-assay SD when a pool of human saliva was assayed repeatedly was ± 0.0013 ng/ml (*n* = 31, mean = 0.0063 ng/ml). Testosterone is reported in units of ng/mL.

### Statistical analyses

Analyses for Aim 1 investigated associations across all development using data collected from all 4 years. Analyses for Aims 2 and 3 were performed separately on data from the first visit only and the last available visit for each subject to investigate the joint mediation effects of cortisol and testosterone on social problems (Aim 2) and aggressive behavior (Aim 3) in early and late pubertal development. Analyses of the last available time point included ages 10–17 (mean = 13.82, SD = 1.64). Prior to conducting the analyses, cortisol and testosterone measurements were log10 transformed. We used type II sum of squares ANOVA to test effects in the mixed effects and ordinary linear regression models, so main effects are tested without their interactions in the model. We also calculated a nonnegative robust effect size index (RESI), denoted by *S*, that is proportional to ½ Cohen’s *d*. Heteroskedasticity consistent standard errors were also used in all regression models, though not in the mediation analyses per limitation in software. All analyses were performed using R (Version 4.3.0). Mixed effects models were fit using the *lme4* package [115], the mediation analyses were done using the *mma* package [116], effect sizes were calculated using the *RESI* package [117], and visualizations created with the *effects* package [118].

#### Aim 1

To examine the trajectory of cortisol and testosterone based on sex (Male, Female) and diagnosis (ASD, TD) over development (Age, Puberty), we fit separate mixed effects models on cortisol and testosterone to see how the trajectories of the hormones changed through development by including a diagnosis by sex by age/pubertal stage interaction, including presence of medication, and BMI as covariates and a random intercept for subject. Age, puberty, and BMI were fit nonlinearly using natural cubic splines with 3 degrees of freedom. Analyses estimating age and puberty effects were fit separately.

#### Aim 2

To investigate the mediation effect of cortisol and testosterone on the diagnostic association with social problems in early and late pubertal development, we performed the following sequence analyses separately using the first time point for each participant and the last available time point for each participant. Mediation analyses were restricted to first and last measurement as software to model the joint mediating effect of cortisol and testosterone was limited to cross-sectional analyses. Prior to investigating the mediation effect, we first investigated whether diagnosis, cortisol, and testosterone were associated with social problems, controlling for all other variables. The outcome model was fit using a linear regression model with age, sex, presence of medication, and BMI as covariates, allowing cortisol, testosterone, age, and BMI to have nonlinear effects via natural cubic splines fit with 3 degrees of freedom. Then, we conducted a multiple mediation analysis investigating the mediating effect of cortisol, testosterone, and their interaction on the association between diagnosis and CBCL Social Problems [116]. We also performed a supplementary analysis using puberty in place of age. To investigate the same effect across all time points, we fit linear mixed effects models on social problems and aggressive behavior over all 4 years of data including both cortisol and testosterone as main variables of interest, while controlling for sex, pubertal stage, BMI, and medication. These models were fit twice, with and without diagnosis to see if the the hormone effect changed. We allowed the hormones, puberty, and BMI effects to be nonlinear with natural cubic splines with 3 degrees of freedom and included a random intercept for subject to account for correlation within-subject.

#### Aim 3

To investigate the influence of the Dual-Hormone Hypothesis in explaining aggressive behavior by differences in hormone profiles in early and late pubertal development we used the same model, approach, and covariates as in Aim 2 with CBCL Aggressive Behavior as the outcome variable.

## Results

### Aim 1

**Fig. 1:**
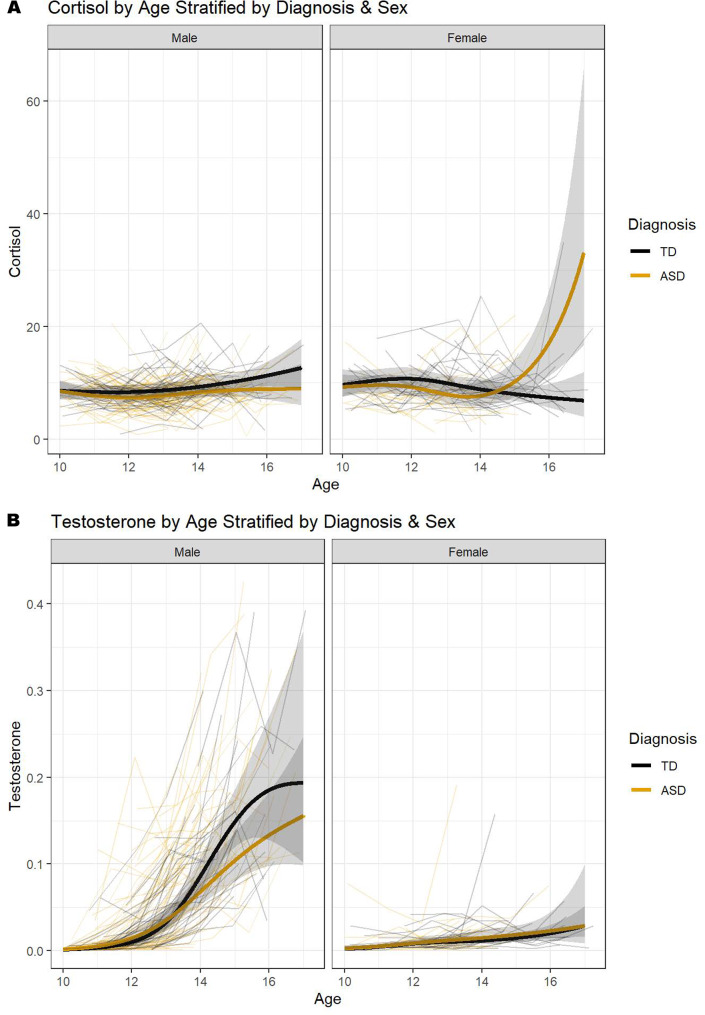
Changes in cortisol (**A**) and testosterone (**B**) for TD and ASD males and females over adolescence. *Note*: Shading show’s 95% confidence intervals and overlap suggests no significant difference

To investigate how hormone levels change through development, we modeled the longitudinal age trajectories of cortisol and testosterone by sex and diagnosis, hypothesizing an increase in both hormones over development. For cortisol, there was a significant diagnosis by sex by age interaction (X^2^ = 15.62, df = 3, *p* = 0.0014, *S* = 0.2446; Table 2), but there was insufficient evidence of this interaction when modelling testosterone as the outcome (X^2^ = 4.31, df = 3, *p* = 0.2297, *S* = 0.0792; Table 3). While there appeared to be no noticeable difference in cortisol trajectories for males between diagnostic groups, autistic females had comparable cortisol levels to TD females, which were relatively stable, during early and mid-adolescence, followed by increasing levels into late-adolescence (Fig. 1a). As expected, we saw a large, significant difference between the sexes in terms of testosterone trajectories over adolescence (X^2^ = 97.66, df = 3, *p* < 0.0001, *S* = 0.6730; Table 3). Females had stable low testosterone that increased slightly over adolescence, whereas males were observed to have rapid increases in testosterone starting at about age 12–13 (Fig. 1b). Additionally, we observed a significant difference in testosterone trajectories between ASD and TD males, with ASD males having significantly stunted testosterone growth compared to TD males (Est = 0.1530, *p* = 0.0130).

**Table 2:** Mixed effects ANOVA table of diagnosis, age, and sex on cortisol

	X^2^	df	Pr(> X^2^)	Effect Size (RESI; S)
Diagnosis	2.2839	1	0.1307	0.0780
Sex	2.0677	1	0.1504	0.0711
Age	5.4820	3	0.1397	0.1085
BMI	4.3045	3	0.2304	0.0786
Medication	0.4445	1	0.5049	0.0000
Diagnosis: Sex	0.0139	1	0.9062	0.0000
Diagnosis: Age	3.9739	3	0.2643	0.0679
Sex: Age	12.2585	3	0.0065	0.2095
Diagnosis: Sex: Age	15.6212	3	0.0014	0.2446

**Table 3:** Mixed effects ANOVA table of diagnosis, age, and sex on testosterone

	X^2^	df	Pr(> X^2^)	Effect Size (RESI; S)
Diagnosis	0.6374	1	0.4247	0.0000
Sex	138.6288	1	0.0000	0.8115
Age	703.3451	3	0.0000	1.8306
BMI	1.9986	3	0.5727	0.0000
Medication	6.3333	1	0.0118	0.1597
Diagnosis: Sex	0.7706	1	0.3800	0.0000
Diagnosis: Age	4.7697	3	0.1895	0.0920
Sex: Age	97.6607	3	0.0000	0.6730
Diagnosis: Sex: Age	4.3120	3	0.2297	0.0792

**Fig. 2:**
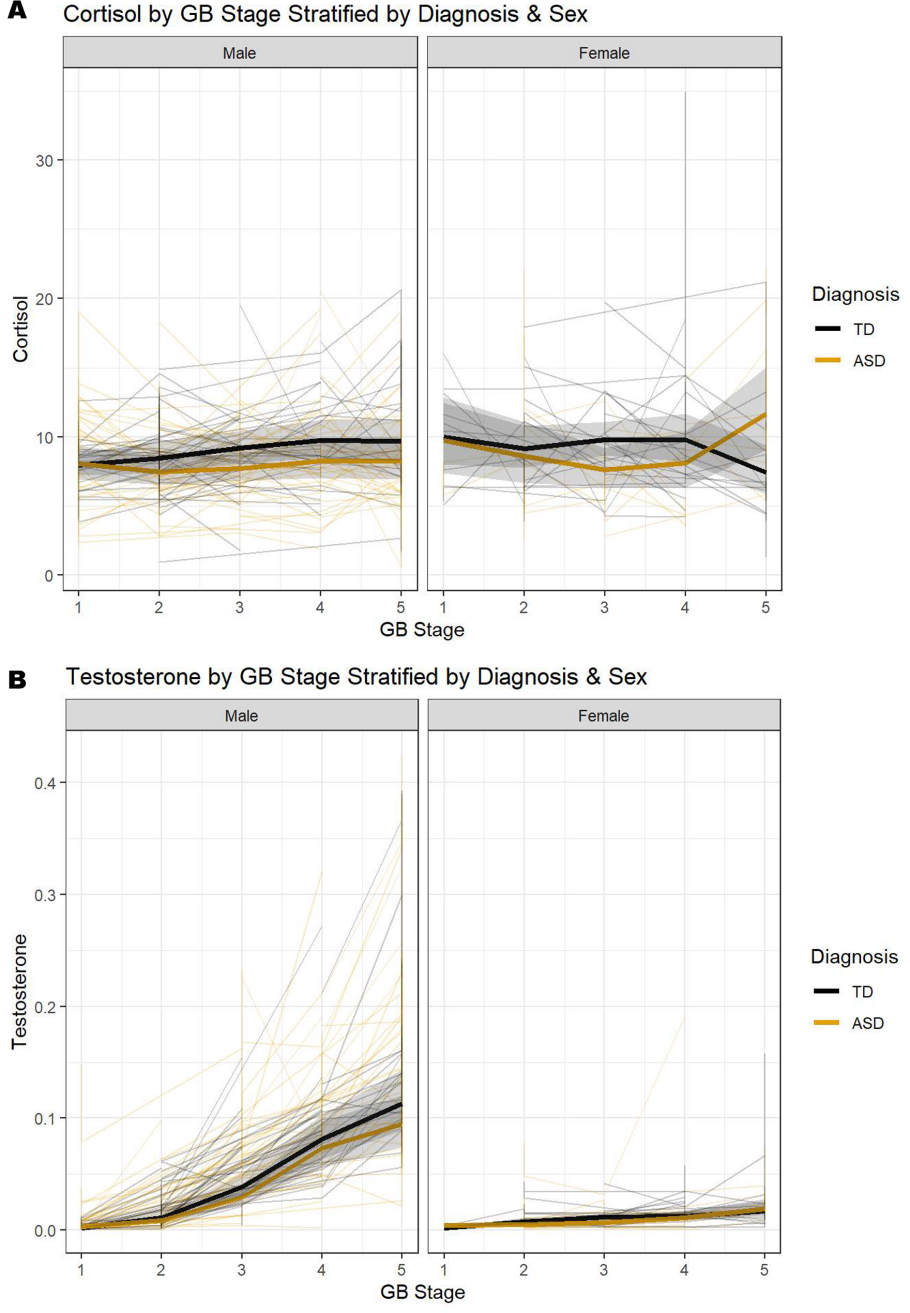
Change in cortisol (**A**) and testosterone (**B**) for TD and ASD males and females through puberty. *Note*: Shading show’s 95% confidence intervals and overlap suggests no significant difference

Results from analyses using pubertal stage roughly mirrored the results as fit with age: there was a significant diagnosis by sex by GB stage interaction for cortisol over pubertal development (X^2^ = 10.92, df = 3, *p* = 0.0122, *S* = 0.1942; Table 4), such that female groups showed comparable levels of cortisol during prepuberty, before levels in autistic females declined during mid-puberty and rise in later pubertal stages; TD females showed relatively stable cortisol levels through mid-puberty before decreasing at later pubertal stages (Fig. 2a). Autistic and TD males retain relatively low cortisol levels throughout puberty (Fig. 2a). Again, there was no evidence for a diagnosis by sex by GB stage interaction with testosterone as the outcome (X^2^ = 2.53, df = 3, *p* = 0.4700, *S* = 0.0000; Table 5). However, there was a significant sex by GB stage interaction (X^2^ = 127.20, df = 3, *p* < 0.0001, *S* = 0.7727; Table 5), showing increasing testosterone for males starting at stage 2, with females retaining low testosterone over puberty (Fig. 2b).

**Table 4:** Mixed effects ANOVA table of diagnosis, GB stage, and sex on cortisol

	X^2^	df	Pr(> X^2^)	Effect Size (RESI; S)
Diagnosis	3.9011	1	0.0483	0.1175
Sex	4.1623	1	0.0413	0.1227
GB Stage	7.4188	3	0.0597	0.1451
BMI	3.9240	3	0.2698	0.0663
Medication	0.1898	1	0.6630	0.0000
Diagnosis: Sex	0.1578	1	0.6912	0.0000
Diagnosis: GB Stage	8.9128	3	0.0305	0.1678
Sex: GB Stage	3.4461	3	0.3278	0.0461
Diagnosis: Sex: GB Stage	10.9224	3	0.0122	0.1942

**Table 5:** Mixed effects ANOVA table of diagnosis, GB stage, and sex on testosterone

	X^2^	df	Pr(> X^2^)	Effect Size (RESI; S)
Diagnosis	0.0912	1	0.7626	0.0000
Sex	151.3751	1	0.0000	0.8503
GB Stage	998.6482	3	0.0000	2.1879
BMI	5.7906	3	0.1223	0.1158
Medication	2.6042	1	0.1066	0.0878
Diagnosis: Sex	0.2223	1	0.6373	0.0000
Diagnosis: GB Stage	25.1826	3	0.0000	0.3266
Sex: GB Stage	127.1964	3	0.0000	0.7727
Diagnosis: Sex: GB Stage	2.5295	3	0.4700	0.0000

### Aim 2

Our second aim was to investigate the mediating effect of cortisol and testosterone on the association between diagnosis and social problems. We first focused on early development and investigated the cortisol by testosterone interaction, their main effects, and the effect of diagnosis in the full model with CBCL Social Problems score as the outcome. There was insufficient evidence for a cortisol by testosterone interaction (X^2^ = 3.50, df = 9, *p* = 0.9412, *S* = 0.0000; Table 6) and a main effect of testosterone (X^2^ = 0.83, df = 3, *p* = 0.8419, *S* = 0.0000; Table 6). However, there were significant effects of diagnosis (X^2^ = 80.72, df = 1, *p* < 0.0001, *S* = 0.5704; Table 6) and cortisol (X^2^ = 14.42, df = 3, *p* = 0.0024, *S* = 0.2159; Table 6) such that higher morning cortisol was associated with lower social problems. To investigate whether testosterone, cortisol, or their combination mediated the association of diagnosis with social problems, we performed a multiple mediation analysis. The total effect of diagnosis aligned with results from the full model, indicating adolescents with ASD were estimated to have a 12.59 higher CBCL Social Problems score than an otherwise identical TD adolescent (CI=[10.08, 15.43], *p* < 0.001; Table 7). There was insufficient evidence of a mediation effect of cortisol (Est = 0.27, CI=[-0.83, 1.24], *p* = 0.6991; Table 7), T (Est = 0.11, CI=[-1.16,0.86], *p* = 0.7739; Table 7), or their joint effect (Est = 0.39, CI=[-1.05, 1.46], *p* = 0.7492; Table 7) on the association between diagnosis and CBCL Social Problems. Together, these results suggest diagnosis and cortisol have unique impact on social problems in adolescence. Analyses in later development found an effect of diagnosis, but no effects cortisol or testosterone (Table S1) and no evidence for hormonal mediation of the diagnostic effect (Table S2). The results were similar using puberty instead of age in the model (Tables S3, S4, & S5). The longitudinal analyses (Tables S9 and S10) showed nonsignificant effects of cortisol (X^2^ = 6.71, df = 3, *p* = 0.0816, *S* = 0.1343) and testosterone (X^2^ = 1.39, df = 3, *p* = 0.7073, *S* = 0.0000) without accounting for diagnosis, as well as when accounting for diagnosis (Cortisol: X^2^ = 6.69, df = 3, *p* = 0.0825, *S* = 0.1338; Testosterone: X^2^ = 1.42, df = 3, *p* = 0.7006, *S* = 0.0000).

**Table 6:** Aim 2 mixed effects ANOVA table of year 1 cortisol and testosterone on CBCL social problems

	X^2^	df	Pr(> X^2^)	Effect Size (RESI; S)
Diagnosis	80.7191	1	0.0000	0.5704
Cortisol	14.4219	3	0.0024	0.2159
Testosterone	0.8316	3	0.8419	0.0000
Age	1.8024	3	0.6144	0.0000
Sex	1.9974	1	0.1576	0.0638
Medication	2.3675	1	0.1239	0.0747
BMI	8.7069	3	0.0335	0.1526
Cortisol: Testosterone	3.4987	9	0.9412	0.0000

**Table 7:** Aims 2 and 3 year 1 cortisol and testosterone mediation effects on CBCL social problems and aggressive behavior

		Estimate	95% CI	*p*
CBCL Social Problems	Total Effect	12.5889	(10.0780,15.4275)	0.0000
	Direct Effect	12.2551	(9.8081,15.3142)	0.0000
	Joint Mediation Effect	0.3938	(-1.0520, 1.4620)	0.7492
	Cortisol Mediation Effect	0.2735	(-0.8335, 1.2430)	0.6991
	Testosterone Mediation Effect	0.1147	(-1.1551, 0.8597)	0.7739
CBCL Aggressive Behavior	Total Effect	8.6977	(6.2039,11.1762)	0.0000
	Direct Effect	8.4357	(5.8894,10.9397)	0.0000
	Joint Mediation Effect	0.1666	(-0.9177, 1.4815)	0.6451
	Cortisol Mediation Effect	0.1975	(-0.3995, 1.2266)	0.3188
	Testosterone Mediation Effect	-0.5027	(-1.9517, 1.3557)	0.7239

### Aim 3

Our final aim was to investigate the mediating effect of cortisol and testosterone on the association between diagnosis and aggressive behavior. We first focused on early pubertal development and investigated the cortisol by testosterone interaction, their main effects, and the effect of diagnosis in the full model with CBCL Aggressive Behavior score as the outcome. There was insufficient evidence for a cortisol by testosterone interaction (X^2^ = 3.00, df = 3, *p* = 0.9642, *S* = 0.0000; Table 8) or main effects of T (X^2^ = 3.72, df = 3, *p* = 0.2936, *S* = 0.0541; Table 8) and C (X^2^ = 3.63, df = 3, *p* = 0.3041, *S* = 0.0508; Table 8). There was a significant effect of diagnosis on CBCL Aggression score (X^2^ = 34.39, df = 1, *p* < 0.0001, *S* = 0.3692; Table 8), which was consistent with the significant total effect from the multiple mediation analysis showing those with ASD have an 8.70 higher score on average than TD adolescents (CI: [6.20, 11.18], *p* < 0.0001; Table 7). Further, there was insufficient evidence for mediating effects of testosterone (Est=-0.5027, CI=[-1.95, 1.36], *p* = 0.7239; Table 7), cortisol (Est = 0.1975, CI=[-0.40, 1.23], *p* = 0.3188; Table 7), or their joint effect (Est = 0.1666, CI=[-0.92, 1.48], *p* = 0.6451; Table 7) on the relationship between diagnosis and CBCL Aggressive Behavior. Together, these results suggest diagnosis has a unique association with aggressive behaviors in adolescence independent of hormonal measurements. The same analyses performed on later pubertal development mirrored results of early pubertal development (Tables S2 & S6). The results were similar using puberty instead of age in the model (Tables S5, S7, & S8). Further, the longitudinal analyses showed no significant cortisol (X^2^ = 2.64, df = 3, *p* = 0.4509, *S* = 0.0000) or testosterone (X^2^ = 1.38, df = 3, *p* = 0.7103, *S* = 0.0000) effect on aggressive behavior without accounting for diagnosis, as well as when accounting for diagnosis (Cortisol: X^2^ = 2.23, df = 3, *p* = 0.5261, *S* = 0.0000; Testosterone: X^2^ = 1.34, df = 3, *p* = 0.7204, *S* = 0.0000). Bivariate correlations for Cortisol and Testosterone with CBCL Aggressive Behaviors and CBCL Social Problems by diagnosis and sex across all four time points is provided in Table S11.

**Table 8:** Aim 3 mixed effects ANOVA table of year 1 cortisol and testosterone on CBCL aggressive behaviors

	X^2^	df	Pr(> X^2^)	Effect Size (RESI; S)
Diagnosis	34.3892	1	0.0000	0.3692
Cortisol	3.6311	3	0.3041	0.0508
Testosterone	3.7180	3	0.2936	0.0541
Age	2.3599	3	0.5011	0.0000
Sex	2.6833	1	0.1014	0.0829
Medication	9.6675	1	0.0019	0.1881
BMI	0.4982	3	0.9193	0.0000
Cortisol: Testosterone	3.0019	9	0.9642	0.0000

## Discussion

The overarching goal of the study was to examine the intersection and developmental course of morning cortisol and testosterone in a large, well-characterized sample of autistic and neurotypical youth. For Aim 1, the trajectory of cortisol and testosterone based on sex (Male, Female) and diagnosis (ASD, TD) over development (Age, Puberty) was explored. Results showed that sex, diagnosis and development play a role in the trajectory of cortisol but not testosterone. Specifically, autistic females had comparable and stable morning cortisol levels to TD females during early and mid-adolescence; however, cortisol levels rose in later adolescence and pubertal stages. Cortisol levels in males across both groups remained stable with only a slight increase over development.

A variety of biobehavioral factors may contribute to the higher cortisol in autistic females. It is possible that the advanced pubertal progression reported in autistic females [22, 23] influences higher cortisol levels as a reflection of maturational changes in the HPA axis. Research with this sample has reported earlier breast development and menses in autistic females [22, 23]; thereby, the higher cortisol may coincide with developmental progress. In healthy adolescent females, menarche or the onset of menses, has been associated with higher peak morning cortisol [119]. Additionally, internalizing state is a relevant consideration since depression has been associated with dysregulation of the HPA axis [120]. In a recent meta-analysis, elevated morning cortisol was shown to be predictive of depression in adolescence [121]. Higher rates of depression have been consistently reported in non-autistic adolescent females compared to males [122, 123] and even higher and earlier rates of depression have been found in autistic females [12, 124, 125]. Further, in the current study, a sex by age interaction was also observed which is consistent with research in healthy controls showing that females compared to males exhibit different normative cortisol values [126]. Taken together, diagnosis (ASD), sex (females) and developmental progression are predictive of morning cortisol levels which is important to replicate as it may have clinical relevance, such as a potential association with internalizing symptoms.

Regarding testosterone, a three-way interaction between diagnosis, sex and age was not observed. However, as expected, there was a large, significant difference between the sexes in terms of testosterone trajectories over adolescence. For females, comparable, stable, and low levels of testosterone increased slightly over the adolescent and pubertal progression similar to findings with neurotypical samples [127, 128]. For males in both groups, higher and steeper slopes were exhibited with a sharp rise in testosterone during middle adolescence starting around 12–13 years of age, which is normative [128]. These sex-based differences are consistent with established gonadal hormone differences, including testosterone, between males and females emerging during puberty and signaling sexual maturation (e.g [31, 32, 129]).

Interestingly, in latter adolescence lower levels of testosterone and flatter slopes were shown for autistic males compared to neurotypical male peers. This finding is similar to an earlier study of serum testosterone in males 12 to 18 years of age in which concentrations were significantly lower in the autism compared to the typically developing control group [89]. In another study examining plasma levels of testosterone in pre- and post-pubertal males with autism, intellectual disability or typical development, there were no significant group differences [88] although the sample sizes for each group were quite small. There were also no significant differences in salivary testosterone in young adult males (mean age 19.5 years) with low, moderate and high levels of autistic traits; however, the authors speculated that the pubertal transition may be associated with lowering or normalizing testosterone levels in autism [130]. It is unclear what may be driving the diagnostic difference in males but factors such as internalizing problems, stress and sleep problems may be explored in future research if such findings are replicated. For example, lower testosterone has been associated with elevated depressive symptoms in post-puberal males [131]. Research also highlights the link between stress exposure and differences in testosterone concentration (e.g [132]), such as lower testosterone reported in individuals diagnosed with post-traumatic stress [133]. It has also been proposed that the lower levels of testosterone may play a role in the pathophysiological profile of autistic males [89]. Future research is needed to replicate and extend these findings and determine their clinical relevance for autism, stress, and internalizing pathology.

The objective of Aim 2 was to investigate the mediation effect of cortisol and testosterone on diagnosis’ effect on the manifestation of social problems. It was hypothesized that diagnostic effects on CBCL-SP [92] would be driven by differences in the cortisol and testosterone profile but this hypothesis was not confirmed. Rather, a significant direct effect of diagnosis on CBCL Social Problems was observed showing youth with ASD have significantly higher social challenges than youth with TD. Moreover, there was a significant effect of cortisol in year 1 with and without the presence of an autism diagnosis. In other words, although it was predicted that diagnostic effects on social problems would be driven by the joint cortisol and testosterone profile, there was a direct effect of diagnosis and a direct effect of cortisol on experiencing social problems regardless of diagnostic status. Even so, the inclusion of diagnosis somewhat diminished the hormone effect and it was not significant using the last time point and was weaker in the full longitudinal analysis. The finding suggests that higher cortisol is associated with social challenges although it may not persist through development. It also suggests that while an ASD diagnosis independently contributes to social difficulty this may be distinct from the impact of cortisol in consideration of age or pubertal status. It is not surprising that ASD diagnosis and cortisol were both associated with social problems. Indeed, a core feature of autism is experiencing notable challenges in social functioning [1]. Moreover, atypical regulation of the HPA axis has been frequently reported in ASD [53–57] including blunted diurnal slope [58, 60].

The third aim explored a hypothesized mediation effect of cortisol and testosterone on diagnosis’ effect on aggressive behavior; however, there was no significant joint mediation effect on the relationship between diagnosis and CBCL Aggressive Behavior. There was, however, a main effect of ASD diagnosis and a main effect of cortisol but only when diagnosis was removed from the model. Children with ASD often engage in aggressive behavior [134, 135]. In a large sample of children 2 to 16 years of age, Hill and colleagues (2014) [135] found that one in four children with ASD exhibited clinically significant scores on the CBCL-Aggressive Behavior scale. It is also relevant to note that morning cortisol has been linked to aggressive behavior [136]. Notably, those on at least one psychotropic medication had slightly higher testosterone. The evidence-base for psychotropic medication use and testosterone levels is mixed, with findings often varying by medication type (see [137] for review). However, psychotropic medication use was further associated with aggressive behaviors, and previous research in youth with autism found that elevated externalizing problems as reported on the CBCL were associated with increased psychotropic medications use [138]. While testosterone was not directly, significantly related to aggressive behaviors, the medication effects on both aggression and testosterone suggest important interactions may exist, which warrants future research more closely examining the relationships between medication use, aggression, and testosterone levels in youth with and without ASD.

While direct effects were demonstrated, the hypothesis that cortisol and testosterone would mediate the relationship with social problems and aggressive behavior was not significant thereby not lending support for the dual-hormone hypothesis. Previous research with various populations has been mixed with some research lending support for the high testosterone/low cortisol and aggressive behavior association [48] while other studies do not show a cortisol and testosterone interaction [50] similar to the current findings. However, it is essential to highlight that most previous studies have been conducted in adult participants with a preponderance of males (e.g [39, 48, 50], and findings in mixed samples show larger effect sizes for men than women [139]. Thus, the extent to which the hypothesis may be relevant during adolescence is understudied suggesting that more research may be warranted.

### Strengths, limitations and future directions

Strengths of the current study include a well-characterized diagnostic and comparison sample, rigorous methodology, employment of a four-year longitudinal design, and cross-comparison of hormonal expression of the HPA and HPG axes and relationship to behavioral profiles. The study, however, is regrettably not fully representative of minoritized individuals (based on race, ethnicity or intellectual profile) limiting generalizability. Attrition of the sample is also acknowledged especially between portions of Y2 and Y3 due to some participants not returning after the initial eligibility visit and confirmation of autism diagnosis as well as the COVID-19 pandemic. Another limitation is that only the immediate morning salivary sampling was used due to lower and undetectable concentration levels of testosterone in younger and female children. It is plausible that hormonal patterns and associations may vary based on diurnal collection times or age of participants.

In conclusion, using a behavioral endocrinology approach, sex-based, hormonal, diagnostic and developmental differences were observed showing that autistic females evidence higher morning cortisol that increase over developmental progression. It is highly plausible that early pubertal onset often observed in autistic females [22, 23] corresponds with advanced HPA maturation. Moreover, due to associations between elevations in both cortisol and depression, this observed pattern may indicate a risk factor for depressive symptomology in autistic females. Testosterone levels in males showed expected sharp increases with maturing age and puberty, although in later stages, autistic males had less increase. It will be essential to identify factors and possible consequences for these lower testosterone levels in autistic males. It is apparent that further study is warranted into understanding the influence of the HPA and HPG in autistic females and males, respectively. Finally, behavioral differences were shown for social problems and aggressive behavior in autism; yet these were not associated with testosterone. Although interrelated hormonal patterns predicting aggressive and social behavior were not found, higher cortisol and autism diagnosis independently and together appear to be associated with social challenges. Collectively, these findings underscore the need to elucidate the biobehavioral patterns that emerge during the complex adolescent transition for autistic youth to determine how they impact clinical and long-term outcomes.

## Electronic supplementary material

Below is the link to the electronic supplementary material.

**Supplementary Material 1**: **Additional file 1**: **Supplemental Tables**: ***Table S1***: Aim 2 Mixed Effects ANOVA Table of Cortisol and Testosterone on CBCL Social Problems at Last Year of Data Collection; ***Table S2***: Aims 2 and 3 Cortisol and Testosterone Mediation Effects on CBCL Social Problems and Aggressive Behavior at Last Year of Data Collection; ***Table S3***: Aim 2 Mixed Effects ANOVA Table of Cortisol and Testosterone on CBCL Social Problems with GB Stage; ***Table S4***: Aim 2 Mixed Effects ANOVA Table of Cortisol and Testosterone on CBCL Social Problems at Last Year of Data Collection with GB Stage; ***Table S5***: Aims 2 and 3 Cortisol and Testosterone Mediation Effects on CBCL Social Problems and Aggressive Behavior at Last Year of Data Collection with GB Stage; ***Table S6***: Aim 2 Mixed Effects ANOVA Table of Cortisol and Testosterone on CBCL Aggressive Behaviors at Last Year of Data Collection; ***Table S7***: Aim 2 Mixed Effects ANOVA Table of Cortisol and Testosterone on CBCL Aggressive Behaviors with GB Stage; ***Table S8***: Aim 2 Mixed Effects ANOVA Table of Cortisol and Testosterone on CBCL Aggressive Behaviors at Last Year of Data Collection with GB Stage; ***Table S9***: Descriptive statistics for cortisol and testosterone at each year by diagnosis and sex; ***Table S10***: Longitudinal ANOVA tables for the CBCL Social Problems with and without diagnosis; ***Table S11***: Bivariate Correlations of Cortisol and Testosterone with CBCL by Subgroup and Time Point.

## Data Availability

Data from this study are shared with the National Database for Autism Research (NDAR) (Collection #2683).

## Abbreviations

ADOS: Autism diagnostic observation schedule
ASD: Autism spectrum disorder
CBCL: Child behavior checklist
GB: Genital/breast stage
HPA: Hypothalamic pituitary adrenal
HPG: Hypothalamic pituitary gonadal
IQ: Intelligence quotient
PH: Pubic hair
TD: Typically developing
WASI: Wechsler abbreviated scale of intelligence
Y: Year
